# Color, Starch Digestibility, and In Vitro Fermentation of Roasted Highland Barley Flour with Different Fractions

**DOI:** 10.3390/foods11030287

**Published:** 2022-01-21

**Authors:** Zixuan Zhao, Jian Ming, Guohua Zhao, Lin Lei

**Affiliations:** 1 College of Food Science, Southwest University, Chongqing 400715, China; zhaozixuan0127@163.com (Z.Z.); food_mj@swu.edu.cn (J.M.); zhaogh@swu.edu.cn (G.Z.); 2 Chongqing Key Laboratory of Speciality Food Co-Built by Sichuan, Chongqing 400715, China

**Keywords:** highland barley flour, particle sizes, in vitro starch digestibility, in vitro fermentation

## Abstract

Highland barley (HB) is commonly milled into flour for direct consumption or further processed with other food formulations. Nevertheless, the association between milling and HB flour properties remains lacking. This work studied the effect of particle sizes (coarse, 250–500 μm; medium, 150–250 μm; fine, <150 μm) on physicochemical and nutritional properties of raw and sand-roasted HB flour. Gelatinization enthalpy decreased with increasing particle sizes of raw HB flour, while no endothermic transitions were observed in sand-roasted flour. Sand roasting destroyed starch granules and decreased short-range molecular order. Starch digestibility increased while total short-chain fatty acids (SCFAs) production decreased with decreasing particle sizes in all samples. The relative crystallinity of sand-roasted HB flour decreased by 80–88% compared with raw samples. Sand roasting raised in vitro starch digestibility, while total SCFAs during in vitro fecal fermentation decreased. Sand-roasted HB flour with particle sizes <150 μm had the highest starch digestibility (94.0%) but the lowest production of total SCFAs (1.89–2.24 mM). Pearson’s correlation analysis confirmed the relationship between the nutritional qualities of HB flour and milling.

## 1. Introduction

Recently, the effects of food processing on the sensory and nutritional qualities of whole grain flours and their finished products have attracted increasing interest. Milling not only influences the interaction between proximate compositions (e.g., lipids and starch granules) and digestive enzymes, but also concentrates unique bioactive compounds (e.g., polyphenols, phytosterols, minerals, and dietary fiber) in the different proportions of the tissues present [1,2,3]. Hard cell walls and microstructures of grains can be destructed differently by milling, leading to different colors, physicochemical properties, and starch digestibility in flours and their finished products [1,4].

Milling, a common method to produce flours with a wide range of particle sizes, causes varied distributions of components, bioavailability of nutrients, and baking properties of grain flours. Until now, milling affects textural and pasting qualities of starch, which are detrimental for finished product properties, such as stability, flow, functionality, texture, mouth feel, and structure [1,5,6]. The starch digestion kinetics of rice, barley, and sorghum flours are affected by the particle sizes [7,8]. Edwards et al. [9] found that fine particles (0.2 mm) of wheat porridges were digested faster than coarse particles (2 mm), leading to a higher glycemic response. Breads made with coarser corn flour (>180 µm) had higher volumes and softer crumbs because of their capacity to hold carbon dioxide during proofing, whereas finer corn flours (<106 µm) were more suitable for cakes [10]. In addition, noodles made from wet-milled flour had a harder texture and lower cooking loss than those made from dry-milled flour [11].

Highland barley (*Hordeum vulgare L. var. nudum hook. f, HB*), one of the varieties of the Gramineae wheat family, is mainly cultivated in highland areas of Tibet, Qinghai, west Sichuan, northwest Yunnan, and southwest Gansu, China. Tsampa, flour milled from sand-roasted HB and consumed with ghee, milk dregs, and sugar, has been a traditional Qinghai–Tibetan staple food for centuries. Due to the low glycemic index (39.4–47.5) of HB starch, it is regarded as an ideal staple food for diabetics [12]. HB is a good source of polyphenols, *β*-glucan, phytosterols, and tocols, which are beneficial for health due to their antioxidant, anti-inflammatory [13], antihyperglycemic [14], antihyperlipidemic [15], and anti-ulcerative colitis activities [16]. Additionally, HB contains more protein but less gluten than other grains. To decrease the risk of wheat-related pathologies (e.g., coeliac disease) while improving the sensory and nutritional qualities of grain-based foods, HB flour is usually mixed with other food formulations by partly substituting wheat or rice flour to make home-made cuisines, such as noodles [17], bread [18], biscuits [19], cookies [20], chapattis [21], rusks [22], and cakes [23]. As mentioned above, milling can affect the sensory and nutritional qualities of HB flour and their finished products made from food formulations which partially replace HB flour with other grains flours. However, limited studies have been reported about the impacts of particle size on physiochemical changes, color, starch digestibility, and fermentation in HB flour.

Therefore, the objective of this work was to study the effects of three particle sizes (coarse, 250–500 μm; medium, 150–250 μm; fine, <150 μm) on changes of physicochemical, morphological, and thermal properties of raw and sand-roasted HB flour, with a focus on in vitro starch digestion and fermentation properties. This study might provide a theoretical basis for understanding the relationship between the utility of HB flour and its acceptable physiochemical and functional qualities when mixed with other food formulations in industrialized production.

## 2. Materials and Methods

### 2.1. Materials and Reagents

HB was obtained from Lhasa, Tibet. Acetic acid ≥98%, propionic acid ≥99%, butyric acid ≥99%, valeric acid ≥99%, and 2-ethylbutyric acid ≥98% were obtained from Aladdin Bio-Chem Technology Co., Ltd., (Shanghai, China). Total glucose oxidase/peroxidase (GOPOD) kit, *β*-glucan (mixed linkage) kit, total dietary fiber kit, and total starch kit were obtained via Megazyme International Ireland, Ltd. (Bray Co., Wicklow, Ireland). α-amylase from porcine pancreas type VI-B (A3176, 11 units/mg) and pepsin from porcine gastric mucosa (P7125, ≥400 units/mg) were obtained via Sigma Chemical Co. (St. Louis, Mo., USA). Pancreatic enzyme (S10031, trypsin ≥4000 units/g, pancreatic α-amylase ≥7000 units/g, and pancreatic lipase ≥4000 units/g) was obtained via the Shanghai yuanye Bio-Technology Co. (Shanghai, China). Other chemicals and reagents used were of analytical grade.

### 2.2. Sand Roasting and Milling

First, 100 g HB (conditioned to 30% moisture content at 4 °C) was mixed with 100 μm fine sand (1:3, *w/w*) and vigorously stirred at 220 ± 5 °C for 30 s using an electromagnetic oven (YFR-01-B, Guangdong Qiaochu Electrical Manufacturing Co., Ltd., Foshan, Guangdong, China). Sand roasting was carefully operated so that it led to maximum expansion of HB grains without burning. Then, sand-roasted HB was immediately separated from hot sand and cooled down to room temperature. Sand-roasted HB was ground into flour by a high-speed multifunction mill (Anhui Hualing Kitchen Equipment Co., Ltd., Maanshan, Anhui, China) and fractionated sequentially into three fractions by sieves of 35, 60, and 100 meshes. The flours were designated as Roasted-35 (coarse, 250–500 μm), Roasted-60 (medium, 150–250 μm), and Roasted-100 (fine, <150 μm), respectively. Raw barley was ground as mentioned above and designated as Raw-35 (coarse, 250–500 μm), Raw-60 (medium, 150–250 μm), and Raw-100 (fine, <150 μm), respectively.

### 2.3. Particle Size Analysis

Particle size distributions (PSDs) of tall flours were determined by a Mastersizer Malvern Hydro2000MU (Malvern Instruments, Worcestershire, UK). HB flour was suspended in circulating distilled water at 2000 rpm. The particle size of HB flour was recorded within the range of 0.02–2000 μm under an obscuration of 10–12%. The particle refractive absorption indexes were 1.52 and 0.1, respectively. The refractive index of water as the dispersant was 1.33. The particle sizes were recorded as the 10th percentile (*d*_10_), 50th or median (*d*_50_), and 90th percentile (*d*_90_), respectively.

### 2.4. Flour Analysis

The crude lipid, crude protein, and ash contents of all flours were analyzed according to AOAC methods 960.39 (2000), 955.04 (2000), and 923.03 (2000) [24]. The contents of total starch, dietary fiber, and *β*-glucan were measured using commercial kits. Starch digested within the first 20 min was designated as rapidly digestible starch (RDS); starch digested during 20–120 min was designated as slowly digestible starch (SDS); starch undigested after 120 min was designated as resistant starch (RS) [25]. The polyphenol contents were determined by the Folin–Ciocalteau method [26]. Color values were determined using a color measurement spectrophotometer UltraScan PRO (Hunter Lab, Reston, VA, USA).

### 2.5. Microscope Observation

Polarized light microscopy images of starch granules were analyzed according to a modified method using a BX53 microscope (Olympus, Tokyo, Japan) with a polarized light accessory. Briefly, 5 mg flour (dry basis) was dispersed in 150 μL of 50% glycerol in a glass vial. A drop of flour suspension was then put onto a slide and observed at 400 × magnification (40 × 10).

For scanning electron microscopy (SEM), all flours were analyzed with a scanning electron microscope Phenom Pro 10,102 at 10 kV (SEM, Phenom-World, Holland). The flour was packed on the plate using a double-faced conductive tape and sputter-coated with a thin film of gold. Nitrogen was used to remove the non-attached flour using a blowing device and observed at 3000× magnification.

### 2.6. Laser Confocal Micro-Raman Spectroscopy

A DXR2 Raman microscopy system was used to acquire laser confocal micro-Raman spectra (Thermo Fisher Scientific, Madison, WI, USA) with an Olympus BX51 microscope (Olympus, Tokyo, Japan). Spectra of 3200–100 cm^−1^ were obtained from three different positions under a 785 nm green diode. As an indicator of the short-range ordered structures in starch [27], the full width at half maximum (FWHM) of the band at 480 cm^−1^ was acquired by Peak Fit v4.12 software.

### 2.7. X-ray Diffraction (XRD)

The crystallinity structures of all flours were scanned at 4–40° (2*θ*) using an X’Pert3 Powder XRD system (PANalytical B.V., Almelo, The Netherlands). The flour was mounted tightly on a round glass cell at a rate of 2°/min under 1600 W (40 kV × 40 mA). The ratio of the crystalline area/the total diffraction area was used for calculating relative crystallinity (RC) by MDI Jade 5.0 software (Material Data, Inc., Livermore, CA, USA).

### 2.8. Differential Scanning Calorimetry (DSC)

A Discovery DSC (TA Instruments, New Castle, DE, USA) was used to determine the thermal properties of all flours. To the precisely weighed sample (3 mg) was added 12 mg distilled water in an aluminum pan, which was then sealed and put at 4 °C. After 24 h of equilibration, the pan was heated from 40 to 100 °C at a rate of 10 °C/min. The gelatinization enthalpy (Δ*H*), onset temperature (*T_O_*), peak temperature (*T_P_*), and conclusion temperature (*T_C_*) were acquired by TRIOS data recording software. An empty aluminum pan was used as the control.

### 2.9. In Vitro Simulated Saliva–Gastrointestinal Digestion

An in vitro simulation of saliva–gastrointestinal digestion was studied according to a previous method with modifications [28]. First, 10 g flour was passed through the simulated in vitro digestion system containing oral (5 min), gastric (120 min), and intestinal (180 min) phases in a water bath vibrator (37 °C) at 120 rpm. During simulated intestinal digestion phase, 1 mL digesta aliquot was obtained and mixed with pure ethanol (4 mL) to terminate the enzymatic reaction at each designated time point (0, 20, 40, 60, 120, and 180 min, respectively). The resulting mixture was then centrifuged at 25 °C, 2151× *g* (TDL-5A centrifuge, Changzhou Yineng Experimental Instrument Factory, Changzhou, Jiangsu, China) for 10 min. The released glucose in the supernatant was analyzed using the Megazyme GOPOD kit and converted into starch by multiplying factor 0.9. The starch digestograms were fixed with the first-order equation as described previously [29]:

*C_t_* = *C_∞_ (*1 − e^−*kt*^) (1)

where *C_t_* = starch digested at time *t*, C_∞_ = starch digested at the reaction end point, and *k* = the first order rate coefficient calculated from the slope of a linear least squares fit of a plot of ln (1 − *C*/*C_∞_*) against *t*.

### 2.10. In Vitro Simulated Fermentation

The digesta obtained from simulated in vitro saliva–gastrointestinal digestion was centrifuged at 25 °C, 2151× *g* for 10 min to obtain the precipitate, which was freeze-dried and then subject to in vitro simulated fecal fermentation [30]. Three healthy volunteers (two females and one male, 21–24 years of age) were the donors of fresh fecal samples, who did not have antibiotic treatments or digestive diseases during the past three months. Firstly, fresh fecal samples obtained from volunteers were diluted by sterilized phosphate buffered saline (0.1 M, pH 7.2) and then filtered to obtain a 10% fecal slurry (*w/v*). Secondly, 100 mg digesta precipitate was dissolved in 9 mL of autoclaved basal nutrient medium (2.0 g/L yeast extract, 2.0 g/L peptone, 0.1 g/L NaCl, 0.04 g/L K_2_HPO_4_, 0.04 g/L KH_2_PO_4_, 0.01 g/L MgSO_4_(H2O)_7_, 0.01 g/L CaCl_2_, 2.0 g/L NaHCO_3_, 0.5 g/L bile salts, 0.5 g/L L-Cysteine-HCL, 0.02 g/L chlorhematin, 1.0 mg/L resazurin, 9.8 mg/L vitamin K, and 2 mL Tween-80). Thirdly, the resultant mixture was thoroughly mixed with fecal slurry (1 mL) and incubated in a thermostatic shaker ZWY-2102C (Zhicheng, Shanghai, China) at 37 °C. Anaerobic condition was kept during in vitro fermentation using an anaerobic chamber (Thermo Fisher Scientific, Madison WI, USA). The fermented samples were obtained at 6, 12, 24, and 48 h, respectively, which were then centrifuged at 25 °C, 2809× *g* for 10 min to collect the supernatant. Test tubes without digesta precipitates were used as blanks.

### 2.11. Determination of pH and Short-Chain Fatty Acids

The pH value of the supernatant obtained from in vitro fecal fermentation was determined using a pH meter (Five Easy, METTLER-TOLEDO, Shanghai, China). Short-chain fatty acids were analyzed according to a previous method with minor modifications [31]. Briefly, the supernatant (0.5 mL) was extracted by acidified 50% ethanol (0.5 mL, pH = 2) with 2-ethylbutyric acid as an internal standard. The mixture was sonicated for 20 min and centrifuged at 4 °C, 9170× *g* (TGL-16.5M high-speed freezing centrifuge, Shanghai Lu Xiangyi Centrifuge Instrument Co., Ltd., Shanghai, China) for 20 min to obtain the supernatant. Short-chain fatty acids were analyzed on a Shimadzu GC-2010 gas chromatograph (Shimadzu, Kyoto, Japan) with a CP-FFAP-CB capillary column (25 m × 0.32 mm × 0.30 μm, Agilent, Santa Clara, CA, USA). Short-chain fatty acids were determined by the retention times of authentic standards and quantified by the amount of 2-ethylbutyric acid.

### 2.12. Data Analysis

Data was presented as means ± standard deviations (*n* = 3). Any significant difference among six groups was analyzed using one-way analysis of variance (ANOVA) followed by post hoc LSD analysis at *p* < 0.05 using IBM SPSS 25.0 software (Chicago, Illinois, USA). The partial correlation coefficients between particle size and starch digestibility and fermentation properties were investigated using SPSS Statistics 25.0 followed by a two-tailed Pearson’ correlation analysis. Asterisks “*” and “**” indicate the different levels of associations significant at *p* < 0.05 and *p* < 0.01, respectively.

## 3. Results and Discussion

### 3.1. Physiochemical Analysis

#### 3.1.1. Particle Size Analysis, Morphological Analysis, and Color Values

**Figure 1:**
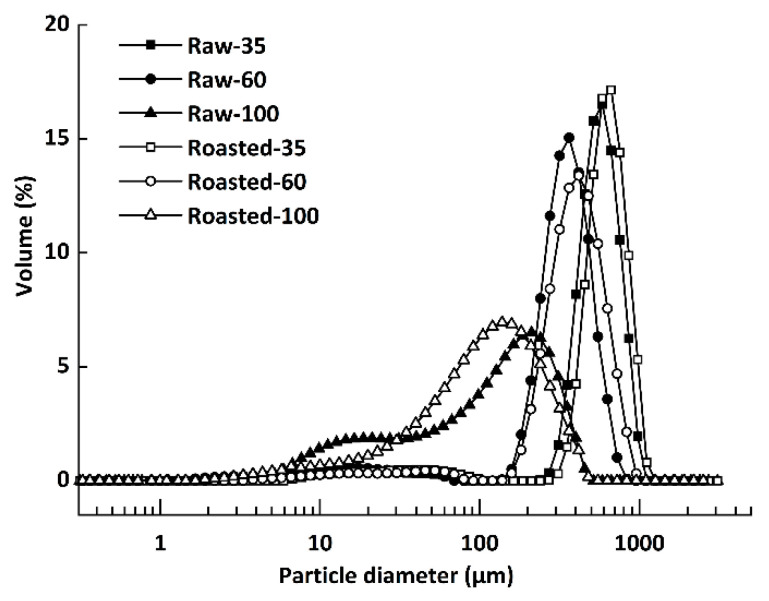
Particle size distribution of raw and sand-roasted highland barley flours. Raw-35, Raw-60, and Raw-100 indicate raw highland barley flour as coarse (250–500 μm), medium (150–250 μm), and fine (<150 μm) fraction, respectively; while Roasted-35, Roasted-60, and Roasted-100 indicate sand-roasted highland barley flour as coarse (250–500 μm), medium (150–250 μm), and fine (<150 μm) fraction, respectively.

The PSD of all flours are presented in Figure 1. Five groups with unimodal PSD patterns were observed, with median particle sizes (*d*_5__0_) of 584 μm (Raw-35), 317 μm (Raw-60), 665 μm (Roasted-35), 367 μm (Roasted-60), and 129 μm (Roasted-100), respectively. However, Raw-100 (*d*_5__0_ = 103 μm) showed a clear bimodal distribution, which represented free starch granules of about 25 μm [32].

**Figure 2:**
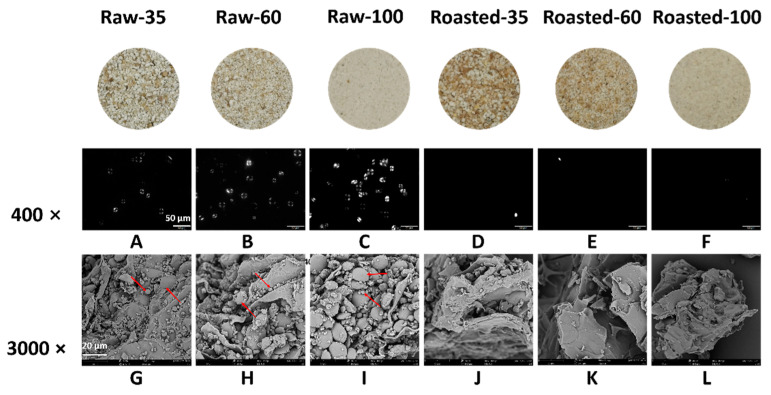
Images of polarized light microscope (PLM) and scanning electron microscope (SEM) of raw and sand-roasted highland barley flours. Raw-35, Raw-60, and Raw-100 indicate raw highland barley flour as coarse (250–500 μm), medium (150–250 μm), and fine (<150 μm) fraction, respectively; while Roasted-35, Roasted-60, and Roasted-100 indicate sand-roasted highland barley flour as coarse (250–500 μm), medium (150–250 μm), and fine (<150 μm) fraction, respectively. The scale bar in the PLM is 50 μm (**A**–**F**) with magnification of 400×; the scale bar in the SEM is 20 μm (**G**–**L**) with magnification of 3000×. The red arrows in the SEM (**G**–**I**) figures present the morphology of starch granules with different particle sizes. (**A**) PLM of Raw-35. (**B**) PLM of Raw-60; (**C**) PLM of Raw-100; (**D**) PLM of Roasted-35; (**E**) PLM of Roasted-60; (**F**) PLM of Roasted-100; (**G**) SEM of Raw-35; (**H**) SEM of Raw-60; (**I**) SEM of Raw-100; (**J**) SEM of Roasted-35; (**K**) SEM of Roasted-60; (**L**) SEM of Roasted-100.

The polarized light microscopic images of all flours are shown in Figure 2. The anisotropic structure between crystalline and amorphous regions in the starch granule presents a typical Maltese cross pattern under polarized light in raw flours (Figure 2A–C). Granular birefringence of Raw-100 appeared stronger than that of Raw-35 and Raw-60 under the same background. This might be due to a greater release of starch from smaller particles. Compared with raw flours (Figure 2A–C), vague contours of gelatinized starch were observed, and the Maltese cross nearly disappeared in sand-roasted flours (Figure 2D–F). This was because sand roasting destroyed the starch crystalline and large granules were susceptible towards gelatinization [33]. Figure 2G–L shows the microstructure of all flours. The circular and elliptical starch granules were entrapped by the cell wall of raw flours (Figure 2G–I), which were disrupted by sand roasting (Figure 2J–L). The lamellar and loose structure in sand-roasted flours (Figure 2J–L) indicated substantial gelatinized starch. This was in accordance with the disappearance of the bright Maltese crosses presented in Figure 2D–F.

The anisotropic structure color values of all samples are shown in Table 1. Lightness (L*) increased as particle size decreased, while redness (a*) and yellowness (b*) showed a reverse trend. This was because of the presence of more bran particles in coarse and medium fractions [34]. Sand roasting significantly decreased lightness value (L*) and increased redness (a*) and yellowness (b*), indicating Maillard reactions caused by the thermal breakdown of starch [35].

#### 3.1.2. Proximate Analysis

The chemical compositions of all samples are demonstrated in Table 1. The contents of crude protein, RS, total dietary fiber (TDF), insoluble dietary fiber (IDF), and polyphenol significantly decreased, while the contents of total starch, RDS, and SDS significantly increased with decreasing particle sizes in both raw and sand-roasted samples. The differences among three particle sizes might be ascribed to different compositions of tissue layers of HB [36]. Sand roasting was in favor of milling bran into smaller fractions and made *β*-glucan-rich fragments evenly distributed in each flour fraction (4.5–4.9%). Raw and sand-roasted samples had similar contents of crude lipids and soluble dietary fiber (SDF) but varied in other compositions. Compared with raw samples, sand roasting significantly raised the contents of RDS and SDS while it reduced the contents of RS, *β*-glucan, total polyphenols (TPs), and free polyphenols (FPs).

#### 3.1.3. Short-Range Ordered Molecular Structure and Crystalline Structure

The FWHM of the Raman band at 480 cm^−1^ in all flours are presented in Table 2. The short-range ordered structure of starch is inversely associated with the FWHM of the Raman band at 480 cm^−1^. Little difference was observed in FWHM of the Raman band at 480 cm^−1^ among raw or sand-roasted samples, indicating that milling barely affected the short-range molecular order. Compared with raw flours (16.57–17.41), sand roasting increased the FWHM of the Raman band at 480 cm^−1^ (18.10–18.88), indicating destroyed crystalline structure and increased amorphous regions of starch.

**Figure 3:**
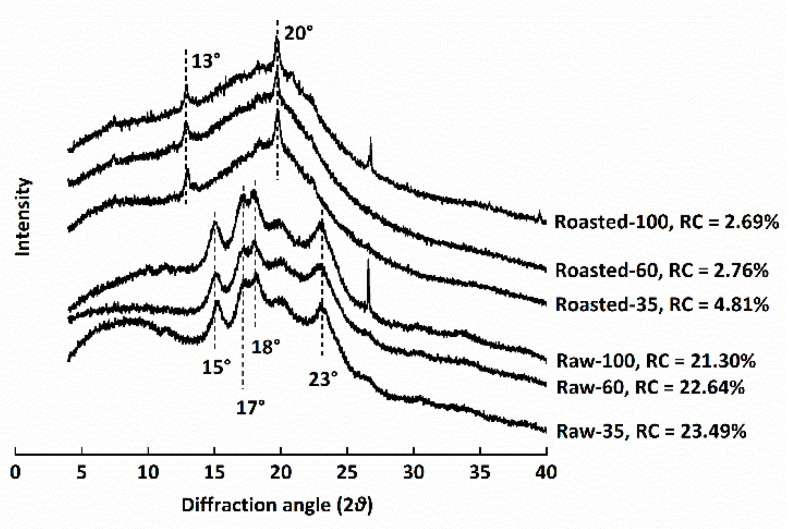
X-ray diffraction patterns of raw and sand-roasted highland barley flours. Raw-35, Raw-60, and Raw-100 indicate raw highland barley flour as coarse (250–500 μm), medium (150–250 μm), and fine (<150 μm) fraction, respectively; while Roasted-35, Roasted-60, and Roasted-100 indicate sand-roasted highland barley flour as coarse (250–500 μm), medium (150–250 μm), and fine (<150 μm) fraction, respectively. RC: relative crystallinity.

The XRD profiles of all flours are shown in Figure 3. The relative crystallinity (RC) of raw flours ranged from 21.30 to 23.49%, similar to a previous study, while the RC of sand-roasted flours decreased to 2.69–4.81%. RC decreased with decreasing particle sizes among either raw or sand-roasted flours. The typical A-type diffraction pattern of starch in raw HB flours, namely, reflection intensities at 15°, 17°, 18°, and 23° (2θ), disappeared after sand roasting. This indicated the full gelatinization of starch granules and the destruction of semi-crystalline starch structures. Compared with raw HB flours, sand-roasted flours presented two peaks at 13° and 20° (2θ) due to the formation of amylose–lipid complexes (V-type) [8].

### 3.2. Thermal Properties

**Figure 4:**
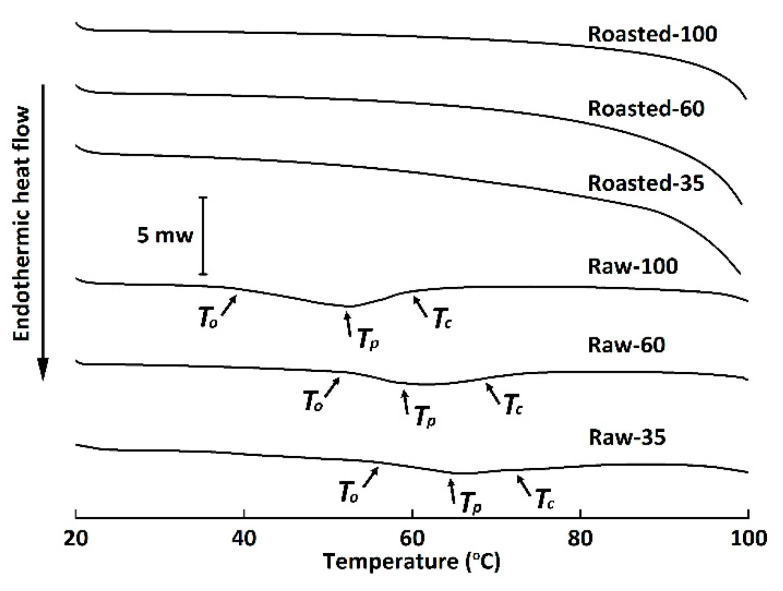
Differential scanning calorimetry thermograms of raw and sand-roasted highland barley flours. Raw-35, Raw-60, and Raw-100 indicate raw highland barley flour as coarse (250–500 μm), medium (150–250 μm), and fine (<150 μm) fraction, respectively; while Roasted-35, Roasted-60, and Roasted-100 indicate sand-roasted highland barley flour as coarse (250–500 μm), medium (150–250 μm), and fine (<150 μm) fraction, respectively. *To*, onset temperature; *Tp*, peak temperature; *Tc*, conclusion temperature.

Starch gelatinization, an endothermic transition, is associated with the dissociation of amylopectin double helices from the ordered structure to an amorphous conformation. The DSC thermograms of all flours are shown in Figure 4. Raw-100 and Raw-60 exhibited significantly lower values of *T_O_*, *T_P_*, and *T_C_* than Raw-35, indicating that starch granules entrapped inside the cell walls of coarse HB flour were more resistant to gelatinization [37]. Our results were in contrast to the findings of Guo et al. [36] and Farooq et al. [8], who reported that milling had no effect on gelatinization temperatures or that lower gelatinization temperatures were observed for coarse fractions. The discrepancy might be ascribed to the different grain varieties, milling techniques, and starch granule sizes. The Δ*H* of Raw-35 significantly decreased in comparison to Raw-100, possibly due to the fact that cell wall encapsulation limited starch swelling and gelatinization [8,36]. No endothermic transitions were observed in sand-roasted HB flour, which demonstrated that starch granules of sand-roasted flours were fully gelatinized [36]. Results were in accordance with the disappeared of bright Maltese crosses (Figure 2D–F) and decreased RC (Figure 3) in our study.

### 3.3. Digestion and Fermentation Properties

#### 3.3.1. Digestion

**Figure 5:**
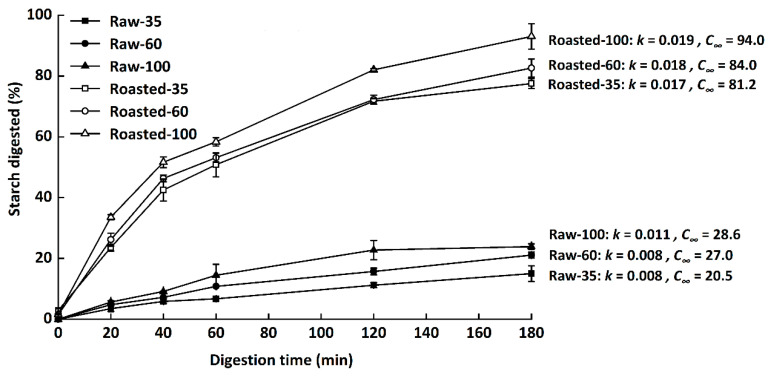
In vitro starch digestion curves of raw and sand-roasted highland barley flours. Raw-35, Raw-60, and Raw-100 indicate raw highland barley flour as coarse (250–500 μm), medium (150–250 μm), and fine (<150 μm) fraction, respectively; while Roasted-35, Roasted-60, and Roasted-100 indicate sand-roasted highland barley flour as coarse (250–500 μm), medium (150–250 μm), and fine (<50 μm) fraction, respectively. *k*, the kinetic constant; *C_∞_*, maximal equilibrium concentration.

Digestograms and values of *k* and *C_∞_* of starch in all samples are presented in Figure 5. The digestibility of starch was presented as the hydrolysis rate of total starch at 0–180 min. *C_∞_* increased with decreasing particle sizes among either raw or sand-roasted flours. Similarly, Farooq et al. [8] found that coarse fractions had more bran particles and smaller surface areas than medium and fine fractions, resulting in reduced water diffusion and enzyme susceptibility. The hydrolysis rate (*k*, 0.008–0.011) and extent (*C_∞_*, 20.5–28.6) of raw flours were much lower than those of sand-roasted samples (*k*, 0.017–0.019; *C_∞_*, 81.2–94.0), demonstrating that sand roasting significantly enhanced in vitro starch digestion rates.

#### 3.3.2. Fermentation

**Figure 6:**
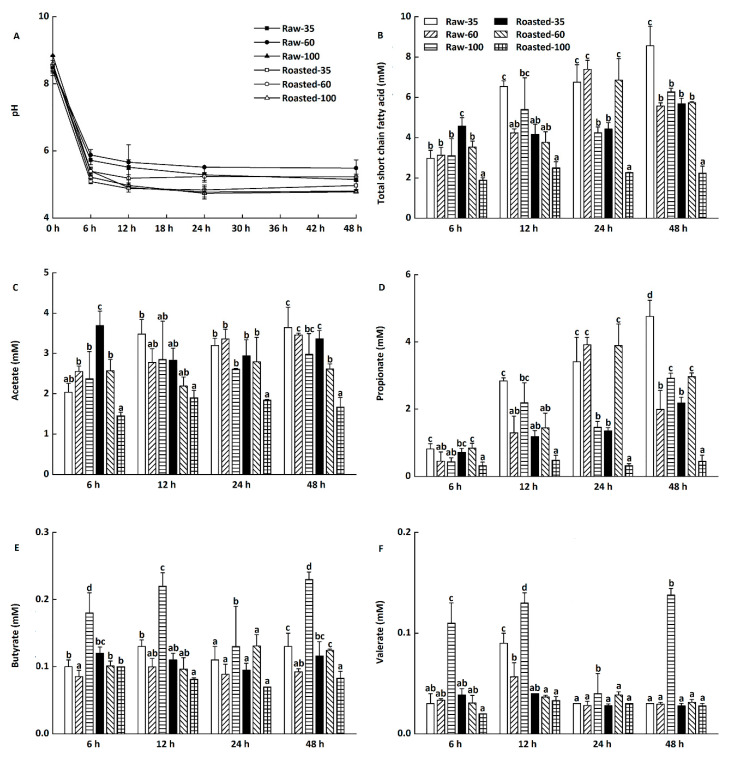
Changes of pH, total short-chain fatty acids (SCFAs), acetate, propionate, butyrate, and valerate during in vitro fermentation of highland barley flours. Raw-35, Raw-60, and Raw-100 indicate raw highland barley flour as coarse (250–500 μm), medium (150–250 μm), and fine (<150 μm) fraction, respectively; while Roasted-35, Roasted-60, and Roasted-100 indicate roasted highland barley flour as coarse (250–500 μm), medium (150–250 μm), and fine (<150 μm) fraction, respectively. a–d: Means with different letters differ significantly at each designated time (*p* < 0.05). (**A**) Changes of pH by time points; (**B**) Changes of total SCFAs by time points; (**C**) Changes of acetate by time points. (**D**) Changes of propionate by time points; (**E**) Changes of butyrate by time points; (**F**) Changes of valerate by time points.

Changes of pH and total short-chain fatty acids of in vitro fermentation in digesta precipitates at 6–48 h are presented in Figure 6A,B, respectively (data not shown for blanks). The initial pH value was around 8.39–8.84 during in vitro fermentation, which decreased sharply to 5.09–5.88 after the first 6 h. The pH value continued to slightly decrease from 6 to 24 h and remained almost unchanged at 4.78–5.49 until 48 h (Figure 6A).

The decreased pH was in agreement with the increased production of total short-chain fatty acids (Figure 6B), which could improve colon health via promoting the growth of *Bifidobacterium* and *Lactobacillus* [38,39]. Compared with raw flours, sand-roasted flours produced lower levels of total short-chain fatty acids at 12–48 h, with Roasted-100 having the lowest level of total short-chain fatty acids. This might be because Roasted-100 had the lowest RS content among all flours (Table 1). RS, known as a dietary fiber, is able to produce short-chain fatty acids and therefore improve bowel health [40]. However, Schlormann et al. [41] found no effect of roasting on short-chain fatty acid concentrations and pH values in barley flakes at 24 h. This might be ascribed to the different substrates, processing techniques, and food structures.

Changes in individual short-chain fatty acid production during in vitro fermentation at 6–48 h are presented in Figure 6C–F. The major short-chain fatty acids were acetate and propionate (Figure 6C,F), followed by butyrate and valerate (Figure 6D,E). Compared with in vitro fermentation at 6 h, there was a considerable increase in the concentrations of propionate in all digesta precipitates at 12–48 h (Figure 6D). Kim and White [42] also found that *β*-glucan produced higher proportions of propionate than lactulose and inulin did during in vitro fermentation. Propionate is beneficial in modulating blood glucose, insulin sensitivity, and lipid homeostasis [43,44]. However, only slight changes in concentrations of acetate, butyrate, and valerate were observed during in vitro fermentation at 6–48 h (Figure 6C,E,F).

### 3.4. Correlation Analysis

The correlations between particle size and parameters related to the digestion and fermentation properties of all samples are shown in Table 3. Starch digestibility can be reflected by the contents of RDS and SDS in food, while RS in food can escape digestion in the small intestine and enters the colon for fermentation [25,35]. RDS was negatively related to particle size (r = −0.89, *p* < 0.05). RS showed a very strong positive association with particle size (r = 0.95, *p* < 0.05). In addition, RS had very strong positive correlations with acetate production.

## 4. Conclusions

Overall, medium and fine particles in HB flour might be responsible for the improved hydrolysis rate and extent of starch. More starch remained undigested in raw coarse flours after 180 min enzymatic digestion, leading to higher in vitro fecal short-chain fatty acids production. Raw HB flour with coarse particle sizes might be developed as a functional component or nutraceutical in formulated foods. This study might provide fundamentals for the milling and application of HB flour in industrialized production.

## Figures and Tables

**Table 1:** Proximate compositions, bioactive compounds, starch fractions (RDS, SDS, and RS), and color values of raw and sand-roasted highland barley flours.

	Raw-35	Raw-60	Raw-100	Roasted-35	Roasted-60	Roasted-100
% of wet basis						
Moisture	10.7 ± 0.1 ^b^	10.9 ± 0.2 ^b^	10.9 ± 0.0 ^b^	5.2 ± 0.9 ^a^	5.7 ± 0.9 ^a^	5.1 ± 0.2 ^a^
% of dry basis						
Crude lipid	3.9 ± 0.6 ^a^	4.0 ± 0.0 ^a^	4.7 ± 0.2 ^a^	4.1 ± 0.3 ^a^	4.4 ± 0.3 ^a^	3.9 ± 0.2 ^a^
Crude protein	7.4 ± 0.2 ^b^	7.0 ± 0.2 ^b^	6.7 ± 0.3 ^a^	7.5 ± 0.3 ^b^	7.4 ± 0.7 ^b^	6.5 ± 0.0 ^a^
Total starch	56.5 ± 0.9 ^a^	60.3 ± 2.1 ^bc^	67.0 ± 1.4 ^d^	53.5 ± 1.4 ^a^	55.2 ± 1.1 ^a^	62.6 ± 2.7 ^c^
RDS	0.0 ± 0.0 ^a^	1.5 ± 0.1 ^ab^	4.1 ± 2.4 ^bc^	6.1 ± 0.1 ^c^	15.0 ± 2.3 ^d^	21.1 ± 0.9 ^e^
SDS	2.8 ± 0.2 ^a^	6.7 ± 0.5 ^b^	13.3 ± 2.0 ^c^	25.7 ± 1.1 ^d^	24.4 ± 0.2 ^d^	30.6 ± 1.3 ^e^
RS	53.7 ± 0.2 ^e^	52.0 ± 0.7 ^e^	49.6 ± 0.4 ^d^	21.7 ± 0.9 ^c^	15.8 ± 2.0 ^b^	10.9 ± 0.4 ^a^
TDF	24.6 ± 0.1 ^b^	24.2 ± 0.9 ^b^	15.8 ± 0.8 ^a^	23.7 ± 0.5 ^b^	25.1 ± 0.4 ^b^	17.9 ± 1.8 ^a^
SDF	7.9 ± 0.1 ^a^	8.8 ± 1.2 ^a^	6.8 ± 0.5 ^a^	7.7 ± 0.5 ^a^	8.2 ± 1.3 ^a^	8.8 ± 1.9 ^a^
IDF	16.7 ± 0.3 ^c^	15.4 ± 0.3 ^a^	9.0 ± 0.3 ^a^	16.1 ± 0.5 ^bc^	16.8 ± 1.0 ^c^	9.1 ± 0.2 ^a^
*β*-glucan	5.5 ± 0.3 ^c^	5.4 ± 0.4 ^c^	2.8 ± 0.1 ^a^	4.9 ± 0.1 ^b^	4.7 ± 0.2 ^b^	4.5 ± 0.2 ^bc^
Polyphenol (mg/100 g dry basis)						
TP	262.6 ± 1.6 ^e^	169.7 ± 13.0 ^c^	148.7 ± 5.0 ^b^	231.0 ± 12.5 ^d^	174.3 ± 4.1 ^c^	122.5 ± 4.2 ^a^
FP	191.5 ± 1.1 ^e^	121.9 ± 11.0 ^c^	96.1 ± 6.3 ^b^	170.8 ± 10.7 ^d^	123.0 ± 2.7 ^c^	76.5 ± 4.3 ^a^
BP	71.1 ± 0.4 ^b^	47.8 ± 2.0 ^a^	52.6 ± 1.8^a^	60.2 ± 12.3 ^ab^	51.3 ± 1.5 ^a^	46.0 ± 0.1 ^a^
Color values						
Lightness (L*)	86.3 ± 1.3 ^c^	94.1 ± 1.2 ^d^	94.7 ± 0.2 ^d^	77.1 ± 1.1^a^	79.6 ± 0.4 ^b^	86.8 ± 0.1 ^c^
Redness (a*)	3.7 ± 0.2 ^b^	3.0 ± 0.1^a^	2.9 ± 0.1 ^a^	6.5 ± 0.3 ^d^	6.2 ± 0.3 ^d^	4.4 ± 0.1 ^c^
Yellowness (b*)	11.2 ± 0.2 ^b^	10.0 ± 0.4 ^a^	9.9 ± 0.0 ^a^	17.0 ± 0.6 ^d^	17.0 ± 0.2 ^d^	14.3 ± 0.1 ^c^

Raw-35, Raw-60, and Raw-100 indicate raw highland barley flour as coarse (250–500 μm), medium (150–250 μm), and fine (<150 μm) fraction, respectively; while Roasted-35, Roasted-60, and Roasted-100 indicate sand-roasted highland barley flour as coarse (250–500 μm), medium (150–250 μm), and fine (<150 μm) fraction, respectively. RDS, rapidly digestible starch; SDS, slowly digestible starch; RS, resistant starch; TDF, total dietary fiber; SDF, soluble dietary fiber; IDF, insoluble dietary fiber; TP, total polyphenol; FP, free polyphenol; BP, bond polyphenol. Means with different letters in a row differ significantly (*p* < 0.05).

**Table 2:** Short-range molecular orders of starch in raw and sand-roasted highland barley flours.

Highland Barley Flour Samples	FWHM of the Band at 480 cm^−1^
Raw-35	17.41 ± 0.43 ^ab^
Raw-60	17.38 ± 0.27 ^ab^
Raw-100	16.57 ± 0.44 ^a^
Roasted-35	18.10 ± 1.80 ^bc^
Roasted-60	18.28 ± 0.59 ^c^
Roasted-100	18.88 ± 0.89 ^c^

Raw-35, Raw-60, and Raw-100 indicate raw highland barley flour as coarse (250–500 μm), medium (150–250 μm), and fine (<150 μm) fraction, respectively; while Roasted-35, Roasted-60, and Roasted-100 indicate sand-roasted highland barley flour as coarse (250–500 μm), medium (150–250 μm), and fine (<150 μm) fraction, respectively. FWHM, full width at half maximum. Means with different letters in a column differ significantly (*p* < 0.05).

**Table 3:** Pearson’s correlation matrix of different parameters of raw and sand-roasted highland barley flours (control variable: sand roasting).

Parameter	PS	FWHM	Crys	Δ*H*	RDS	SDS	RS	SCFAs	Acetate	Propionate	Butyrate	Valerate
PS	1.00											
FWHM	−0.02	1.00										
Crys	0.94 *	0.14	1.00									
Δ*H*	−0.66	−0.56	−0.63	1.00								
RDS	0.89 *	0.39	−0.84	0.24	1.00							
SDS	−0.83	−0.30	−0.71	0.79	0.57	1.00						
RS	0.93 *	−0.32	0.86	−0.34	−0.99 **	−0.65	1.00					
SCFAs	0.80	−0.35	0.56	−0.54	−0.71	−0.73	0.76	1.00				
Acetate	0.92 *	−0.31	0.83	−0.33	0.98 **	−0.70	0.99 **	0.77	1.00			
Propionate	0.66	−0.26	0.39	−0.60	−0.48	−0.69	0.56	0.96 **	0.57	1.00		
Butyrate	−0.22	−0.88 *	−0.38	0.47	−0.04	0.49	−0.02	0.23	−0.05	0.26	1.00	
Valerate	−0.56	−0.76	−0.63	0.71	0.25	0.78	−0.33	−0.16	−0.36	−0.11	0.92 *	1.00

PS, particle size; FWHM, full width at half maximum; Crys, crystallinity; ΔH, gelatinization enthalpy; RDS, rapidly digestible starch; SDS, slowly digestible starch; RS, resistant starch; SCFAs, total short chain fatty acids; *, significant difference at *p* < 0.05; **, significant difference at *p* < 0.01.

## Data Availability

Data made available upon reasonable request to the corresponding author.

## References

[1] Gu Z., Jiang H., Zha F., Manthey F., Rao J., Chen B. (2021). Toward a comprehensive understanding of ultracentrifugal milling on the physicochemical properties and aromatic profile of yellow pea flour. Food Chem..

[2] Martin-Garcia B., Pasini F., Verardo V., Gomez-Caravaca A.M., Marconi E., Caboni M.F. (2019). Distribution of free and bound phenolic compounds in buckwheat milling fractions. Foods.

[3] Martín-García B., Verardo V., Diaz de Cerio E., Razola-Díaz M.D.C., Messia M.C., Marconi E., Gómez-Caravaca A.M. (2021). Air classification as a useful technology to obtain phenolics-enriched buckwheat flour fractions. LWT.

[4] Roman L., Gomez M., Li C., Hamaker B.R., Martinez M.M. (2017). Biophysical features of cereal endosperm that decrease starch digestibility. Carbohydr. Polym..

[5] Drakos A., Kyriakakis G., Evageliou V., Protonotariou S., Mandal I., Ritzoulis C. (2017). Influence of jet milling and particle size on the composition, physicochemical and mechanical properties of barley and rye flours. Food Chem..

[6] Liu C., Liu L., Li L., Hao C., Zheng X., Bian K., Zhang J., Wang X. (2015). Effects of different milling processes on whole wheat flour quality and performance in steamed bread making. LWT.

[7] Al-Rabadi G.J., Torley P.J., Williams B.A., Bryden W.L., Gidley M.J. (2012). Particle size heterogeneity in milled barley and sorghum grains: Effects on physico-chemical properties and starch digestibility. J. Cereal Sci..

[8] Farooq A.M., Li C., Chen S., Fu X., Zhang B., Huang Q. (2018). Particle size affects structural and in vitro digestion properties of cooked rice flours. Int. J. Biol. Macromol..

[9] Edwards C.H., Grundy M.M., Grassby T., Vasilopoulou D., Frost G.S., Butterworth P.J., Berry S.E., Sanderson J., Ellis P.R. (2015). Manipulation of starch bioaccessibility in wheat endosperm to regulate starch digestion, postprandial glycemia, insulinemia, and gut hormone responses: A randomized controlled trial in healthy ileostomy participants. Am. J. Clin. Nutr..

[10] de la Hera E., Talegon M., Caballero P., Gomez M. (2013). Influence of maize flour particle size on gluten-free breadmaking. J. Sci. Food Agric..

[11] Heo S., Lee S.M., Shim J.-H., Yoo S.-H., Lee S. (2013). Effect of dry- and wet-milled rice flours on the quality attributes of gluten-free dough and noodles. J. Food Eng..

[12] Moza J., Gujral H.S. (2016). Starch digestibility and bioactivity of high altitude hulless barley. Food Chem..

[13] Ge X., Jing L., Zhao K., Su C., Zhang B., Zhang Q., Han L., Yu X., Li W. (2021). The phenolic compounds profile, quantitative analysis and antioxidant activity of four naked barley grains with different color. Food Chem..

[14] Zheng B., Zhong S., Tang Y., Chen L. (2020). Understanding the nutritional functions of thermally-processed whole grain highland barley in vitro and in vivo. Food Chem..

[15] Deng N., He Z., Guo R., Zheng B., Li T., Liu R.H. (2020). Highland barley whole grain (*Hordeum vulgare* L.) ameliorates hyperlipidemia by modulating cecal microbiota, miRNAs, and AMPK pathways in leptin receptor-deficient db/db mice. J. Agric. Food Chem..

[16] Chen M., Tian S., Li S., Pang X., Sun J., Zhu X., Lv F., Lu Z., Li X. (2021). *β*-Glucan extracted from highland barley alleviates dextran sulfate sodium-induced ulcerative colitis in C57BL/6J mice. Molecules.

[17] Zhao B., Shang J., Wang L., Liu L., Tong L., Zhou X., Wang S., Zhang Y., Zhou S. (2020). Evaluation of ingredient mixing procedure on quality characteristics of noodles enriched with half hulless barley flour. Int. J. Food Sci. Technol..

[18] Cakir E., Arici M., Durak M.Z. (2021). Effect of starter culture sourdough prepared with *Lactobacilli* and *Saccharomyces cerevisiae* on the quality of hull-less barley-wheat bread. LWT.

[19] Martínez-Subirà M., Romero M.P., Puig E., Macià A., Romagosa I., Moralejo M. (2020). Purple, high *β*-glucan, hulless barley as valuable ingredient for functional food. LWT.

[20] Deng X.Q., Pan Z.F., Li Q., Deng G.B., Long H., Tashi N., Zhao Y., Yu M.Q. (2019). Nutritional components, in vitro digestibility, and textural properties of cookies made from whole hull-less barley. Cereal Chem..

[21] Narwal S., Kumar D., Sheoran S., Verma R.P.S., Gupta R.K. (2017). Hulless barley as a promising source to improve the nutritional quality of wheat products. J. Food Sci..

[22] Punia S., Sandhu K.S., Kaur M. (2020). Quantification of phenolic acids and antioxidant potential of wheat rusks as influenced by partial replacement with barley flour. J. Food Sci..

[23] Ruan Z., Zhang C., Sun-Waterhouse D., Li B.-S., Li D.-D. (2019). Chiffon cakes made using wheat flour with/without substitution by highland barley powder or mung bean flour: Correlations among ingredient heat absorption enthalpy, batter rheology, and cake porosity. Food Bioproc. Technol..

[24] AOAC (2000). Official Method of Analysis.

[25] Englyst H.N., Kingman S.M., Cummings J.H. (1992). Classification and measurement of nutritionally important starch fractions. Eur. J. Clin. Nutr..

[26] Srivastava A., Greenspan P., Hartle D.K., Hargrove J.L., Amarowicz R., Pegg R.B. (2010). Antioxidant and anti-inflammatory activities of polyphenolics from Southeastern, U.S. range blackberry cultivars. J. Agric. Food Chem..

[27] Wang S., Li C., Zhang X., Copeland L., Wang S. (2016). Retrogradation enthalpy does not always reflect the retrogradation behavior of gelatinized starch. Sci. Rep..

[28] Minekus M., Alminger M., Alvito P., Ballance S., Bohn T., Bourlieu C., Carriere F., Boutrou R., Corredig M., Dupont D. (2014). A standardised static in vitro digestion method suitable for food—An international consensus. Food Funct..

[29] Goñi I., Garcia-Alonso A., Saura-Calixto F. (1997). A starch hydrolysis procedure to estimate glycemic index. Nutr. Res..

[30] Li W., Wang K., Sun Y., Ye H., Hu B., Zeng X. (2015). Influences of structures of galactooligosaccharides and fructooligosaccharides on the fermentation in vitro by human intestinal microbiota. J. Funct..

[31] Zhao G., Nyman M., Jonsson J.A. (2006). Rapid determination of short-chain fatty acids in colonic contents and faeces of humans and rats by acidified water-extraction and direct-injection gas chromatography. Biomed. Chromatogr..

[32] Protonotariou S., Drakos A., Evageliou V., Ritzoulis C., Mandala I. (2014). Sieving fractionation and jet mill micronization affect the functional properties of wheat flour. J. Food Eng..

[33] Langenaeken N.A., De Schepper C.F., De Schutter D.P., Courtin C.M. (2019). Different gelatinization characteristics of small and large barley starch granules impact their enzymatic hydrolysis and sugar production during mashing. Food Chem..

[34] Sakhare S.D., Inamdar A.A., Soumya C., Indrani D., Rao G.V. (2014). Effect of flour particle size on microstructural, rheological and physico-sensory characteristics of bread and south Indian parotta. J. Food Sci..

[35] Dutta H., Mahanta C.L., Singh V., Das B.B., Rahman N. (2016). Physical, physicochemical and nutritional characteristics of Bhoja chaul, a traditional ready-to-eat dry heat parboiled rice product processed by an improvised soaking technique. Food Chem..

[36] Guo P., Yu J., Wang S., Wang S., Copeland L. (2018). Effects of particle size and water content during cooking on the physicochemical properties and in vitro starch digestibility of milled durum wheat grains. Food Hydrocoll..

[37] Zhou W., Song J., Zhang B., Zhao L., Hu Z., Wang K. (2019). The impacts of particle size on starch structural characteristics and oil-binding ability of rice flour subjected to dry heating treatment. Carbohydr. Polym..

[38] Ahmed J., Taher A., Mulla M.Z., Al-Hazza A., Luciano G. (2016). Effect of sieve particle size on functional, thermal, rheological and pasting properties of Indian and Turkish lentil flour. J. Food Eng..

[39] Chen G., Xie M., Wan P., Chen D., Ye H., Chen L., Zeng X., Liu Z. (2018). Digestion under saliva, simulated gastric and small intestinal conditions and fermentation in vitro by human intestinal microbiota of polysaccharides from Fuzhuan brick tea. Food Chem..

[40] Zhou D., Ma Z., Hu X. (2021). Isolated pea resistant starch substrates with different structural features modulate the production of short-chain fatty acids and metabolism of microbiota in anaerobic fermentation in vitro. J. Agric. Food Chem..

[41] Schlormann W., Atanasov J., Lorkowski S., Dawczynski C., Glei M. (2020). Study on chemopreventive effects of raw and roasted beta-glucan-rich waxy winter barley using an in vitro human colon digestion model. Food Funct..

[42] Kim H.J., White P.J. (2009). In vitro fermentation of oat flours from typical and high beta-glucan oat lines. J. Agric. Food Chem..

[43] Miyamoto J., Watanabe K., Taira S., Kasubuchi M., Li X., Irie J., Itoh H., Kimura I. (2018). Barley beta-glucan improves metabolic condition via short-chain fatty acids produced by gut microbial fermentation in high fat diet fed mice. PLoS ONE.

[44] Pingitore A., Chambers E.S., Hill T., Maldonado I.R., Liu B., Bewick G., Morrison D.J., Preston T., Wallis G.A., Tedford C. (2017). The diet-derived short chain fatty acid propionate improves beta-cell function in humans and stimulates insulin secretion from human islets in vitro. Diabetes Obes. Metab..

